# L'Électrothérapie: Journal d'Électricité médicale

**Published:** 1888-09

**Authors:** 


*L'Électrothérapie: Journal d'Électricité médicale.* Paris: 11 Rue de Mogador.

This is a monthly journal devoted to the study of Electricity as applied to medicine, published under the editorship of Dr. Léon Danion, with several co-editors. The seventh number, dated July, 1888 (the only one we have received), is chiefly made up of two long papers in large print, the first on the treatment by electrolysis of hypertrophy and of tumours of the prostate, by Dr. Leopold Casper. After some preliminary experiments on animals, he has devised a method by which he inserts the negative electrode into the prostate from the rectum, the positive being a broad, flat electrode, placed on the abdomen. The negative electrode consists of a long needle, insulated except at its extremity. The author seems to have met with a fair measure of success in the few cases already treated. The method is a rational one, and it may be anticipated that some procedure of this kind will be of great service when further experience has enabled us to select the cases suitable for it, and to perfect the mode of operation. The second paper, by Dr. Ladame of Geneva, on "the electrical treatment of sexual troubles in man," gives an exhaustive history of the methods hitherto carried out for the relief of the different disorders comprised under this head, with the particular plan of electrical treatment favoured by the author in each. As might be expected, apart from the mental effect in some of the neuroses obtained by passing an interrupted or constant current through the affected parts, the results attained have not been particularly gratifying. The rest of the periodical is in small print, and contains, *inter alia*, an account of a new and improved apparatus for the use of statical electricity, and of the various electric lamps used for the examination of the eye, throat, bladder, &c., with the special advantages or disadvantages of each. There are some abstracts, gathered from contemporary medical literature, of papers on the electrical treatment of various disorders, and a few abstracts of interesting and important papers on general medical subjects.

We doubt very much whether there is any ultimate gain to be derived from the study of disease from the point of view of its accessibility to treatment by electricity; but if a paper with this special object in view is required, this one will answer the purpose as well as any other.

